# Expression of OsCAS (Calcium-Sensing Receptor) in an *Arabidopsis* Mutant Increases Drought Tolerance

**DOI:** 10.1371/journal.pone.0131272

**Published:** 2015-06-22

**Authors:** Xin Zhao, Mengmeng Xu, Rongrong Wei, Yang Liu

**Affiliations:** College of Life Science, Capital Normal University, Beijing, People's Republic of China; University of Hyderabad, INDIA

## Abstract

The calcium-sensing receptor (CaS), which is localized in the chloroplasts, is a crucial regulator of extracellular calcium-induced stomatal closure in *Arabidopsis*. It has homologs in *Oryza sativa* and other plants. These sequences all have a rhodanese-like protein domain, which has been demonstrated to be associated with specific stress conditions. In this study, we cloned the *Oryza sativa* calcium-sensing receptor gene (*OsCAS*) and demonstrated that OsCAS could sense an increase of extracellular Ca^2+^ concentration and mediate an increase in cytosolic Ca^2+^ concentration. The *OsCAS* gene was transformed into an *Arabidopsis CaS *knockout mutant (Salk) and overexpressed in the transgenic plants. OsCAS promoted stomatal closure. We screened homozygous transgenic *Arabidopsis *plants and determined physiological indices such as the oxidative damage biomarker malondialdehyde (MDA), relative membrane permeability (RMP), proline content, and chlorophyll fluorescence parameters, after 21 days of drought treatment. Our results revealed lower RMP and MDA contents and a higher Proline content in transgenic *Arabidopsis* plants after drought stress, whereas the opposite was observed in Salk plants. With respect to chlorophyll fluorescence, the electron transport rate and effective PSII quantum yield decreased in all lines under drought stress; however, in the transgenic plants these two parameters changed fewer and were higher than those in wild-type and Salk plants. The quantum yield of regulated energy dissipation and nonregulated energy dissipation in PSII were higher in Salk plants, whereas these values were lower in the transgenic plants than in the wild type under drought stress. The above results suggest that the transgenic plants showed better resistance to drought stress by decreasing damage to the cell membrane, increasing the amount of osmoprotectants, and maintaining a relatively high photosynthetic capacity. In conclusion, OsCAS is an extracellular calcium-sensing receptor that helps to compensate for the absence of CaS in *Arabidopsis* and increases the drought stress tolerance of transgenic plants.

## Introduction

Water is vital for plant growth and development, and water deficit stress, permanent or temporary, limits the growth and distribution of natural vegetation and the performance of cultivated plants to a greater extent than any other environmental factor [1]. Biological membranes are the first target of many abiotic stresses, and maintenance of the integrity and stability of membranes under water stress is a major component of drought tolerance in plants [2]. Malondialdehyde (MDA) and relative membrane permeability (RMP), products of membrane lipid peroxidation and cell membrane damage, have been considered as indicators of oxidative damage [3,4,5]. Synthesis of stress proteins is a ubiquitous response to cope with prevailing stressful conditions, including water deficit [6]. Previous studies have shown that plants could increase their resistance to drought by accumulating a high level of proline [7]. Photosynthetic capacity is progressively reduced by stomatal closure in response to water deficit stress. Consequently, saturated photosynthesis requires a lower light intensity, and excess of light energy cannot be used for photosynthesis; this increases the susceptibility of plants to photoinhibition [5,8,9]. Chlorophyll fluorescence measurements have been widely used to study the response of plants to environmental stress [8,10,11,12]. Effective PSII quantum yield [Y(II)] is defined as the proportion of absorbed quanta converted into chemically fixed energy by the photochemical charge separation at PSII reaction centers. Y(NPQ), the quantum yield of regulated energy dissipation in PSII, reflects the downregulation of PSII as a protective mechanism against excess light intensity. Y(NO), the quantum yield of non-regulated energy dissipation in PSII, is indicative of plants having serious problems coping with incident radiation [13,14,15]. The electron transport rate (ETR) can be measured using pulse amplitude-modulated (PAM) fluorimetry during rapid light curve experiments and used to assess the physiological state of the plant [16,17].

The calcium-sensing receptor (CaS) is a chloroplast protein localized in the thylakoid membrane of *Arabidopsis* [18]. The N-terminal acidic calcium-binding region of CaS is exposed to the stromal side of the membrane, and a rhodanese domain is present at the C terminus [19]. CaS in *Oryza sativa* (OsCAS) is highly similar to CaS in *Arabidopsis* (AtCaS). Both proteins have a rhodanese domain at the C terminus [7,20]. This domain is also found in phosphatases, and a variety of proteins such as sulfide hydrogenases and cyanates are deemed to be stress responsive proteins. We therefore speculated that *OsCAS* plays a very important role in resistance to environmental stresses [20]. Previous studies have shown that *AtCaS* could adjust the opening and closing of stomata [18,19]. In general, stomatal closure is related to drought tolerance in plants. Therefore, we believed that there was a close association between *OsCAS* and drought resistance. In this study, we transformed the *OsCAS* gene into the *Arabidopsis thaliana CaS* knockout mutant (Salk). This mutant had T-DNA inserted in the intron region of the *CaS* gene and was identified using immunoblot analysis of isolated thylakoids with *CaS*-specific antibodies, as described by Vainonen [20]. In addition, we screened transgenic homozygous lines to analyze drought resistance characteristics in order to understand the novel functions of *OsCAS*.

## Materials and Methods

### Plant materials and growth conditions

Seeds of the *Arabidopsis* ecotype *Columbia* 0 (Col-0) and *Oryza sativa* Nipponbare were obtained from our laboratory stock. Salk seeds were kindly provided by Zhenming Pei (Duke University, Durham, NC, USA). Col-0, Salk, and transgenic plants were grown either on 1/2 Murashige and Skoog (MS) medium or in 1:1 (v/v) soil/vermiculite mixture under the following conditions: 100 μmol photons·m^-2^·s^-1^ light intensity, 16-h photoperiod, 22°C, and 70% relative humidity. Rice seeds were germinated in water and were then grown in a 1:1(v/v) soil/vermiculite mixture under the following conditions: 200 μmol photons·m^-2^·s^-1^ light intensity, 12-h photoperiod, 25°C, and 70–75% relative humidity.

### Construction of the pcDNA_3_-OsCAS vector and transfection into human embryonic kidney (HEK293) cells

Total RNA of rice seedling leaves was extracted using Trizol reagent (Invitrogen Inc., Gaithersburg, MD, USA). After RNase-free DNase I treatment, 1 μg of total RNA was used to synthesize cDNA using SuperScript III reverse transcriptase (TaKaRa Bio Inc., Dalian, China). Specific primers were used in a polymerase chain reaction (PCR) to amplify the 1164-bp full-length cDNA of *OsCAS* by using LA-Taq DNA Polymerase (TaKaRa), according to the manufacturer’s protocols. Primer pairs (1) and (2) used for PCR, designed using Primer Premier 5.0, are shown in S1 Table. PCR products were purified and cloned into a pcDNA_3_ vector. HEK293 cells were then transiently transfected with empty pcDNA_3_ vector, OsCAS, and *Arabidopsis* CAS. Transfected cells were screened for CICI (Ca^2+^_o_-induced [Ca^2+^]_i_ increases) using ratiometric imaging of the fluorescent Ca^2+^-sensitive dye Fura-2 [21].

### Construction of a plant expression vector, plant transformation, and selection of homozygous transgenic plants

The method used to prepare cDNA was the same as described above. PCR products were purified and cloned into pEASY-T vectors (TaKaRa Bio Inc., Dalian, China) for sequencing (Sangon Biotech., Shanghai, China). After sequencing, the full-length *OsCAS* sequence was inserted between the *Spe*I and *Nco*I sites of the pCAMBIA-1302 vector under the control of the cauliflower mosaic virus (CaMV) 35S promoter (S1 Fig).

The resulting 35S::*OsCAS*–GFP vector construct was introduced into *Agrobacterium tumefaciens* strain GV3101 using the freeze-thaw method. Positive clones were used to transform the Salk plants by flower dipping [22]. Positive transgenic *Arabidopsis* plants were selected on 1/2 MS medium containing hygromycin (25 mg/L). First, we selected 15 seedlings from T_2_ generation plants, among which the segregation ratio of positive plants to negative plants was 3:1 (with Chi-square test, including 1000 seedlings), and transferred these into soil. The seeds of single lines (T_3_ seeds from T_2_ Plants) were harvested. We then selected four lines (L1, L2, L3, L4) from the T_3_ generation plants, among which 200 seedlings all grew true leaves and roots. The four lines were stable homozygotes and used for further analyses.

### Identification of transgenic plants

Total DNA of transgenic plants (L1, L2, L3, and L4), Col-0, and Salk, were extracted using the CTAB method [23]. The PCR reactions, using primer pair (1) (S1 Table) for *OsCAS* amplification and hygromycin-specific primer pair (2) (S1 Table), were carried out according to the following conditions. For *OsCAS* amplification, the PCR conditions were 3 min at 94°C; 30 cycles of 30 s at 94°C, 30 s at 62°C, 90 s at 72°C; and a final extension for 10 min at 72°C. For hygromycin identification, the PCR conditions were 3 min at 94°C; 30 cycles of 30 s at 94°C, 30 s at 56°C, and 1 min at 72°C; and a final extension for 10 min at 72°C. Transgenic plants (L1, L2, L3, and L4) were identified using the abovementioned method, and plasmid DNA was used as the control.

Analysis of gene expression levels was performed using a Bio-Rad iQ5 Real-time PCR Detection System (Bio-Rad, Laboratories, United States) with SYBR Premix Ex Taq (TaKaRa), according to the manufacturer’s protocols. Primers pairs (3), (4), and (5) used for qRT-PCR are shown in S1 Table.

### Stomatal aperture measurement

Stomatal aperture measurements were performed as described by Pei et al. [24], with some modifications. Rosette leaves from 3-week-old soil-grown plants were detached and floated on incubation buffer (10 mM MES; Sigma-Aldrich) for 2 h in 100 μmol m^-2^ s^-1^ light at 22–25°C. After 2 h, Ca^2+^ concentration of the buffer was increased to 2 mM, and the leaves were incubated for an additional 2 h. After the incubation period, epidermal strips of the leaves were observed using a Leica DMRE microscope (Leica Microsystems, Wetzlar, Germany).

### Drought stress treatment

All seeds were surface-sterilized and germinated on 1/2MS medium. After 10 days, uniform and healthy seedlings were selected and transferred to pots (8 cm × 10 cm) that contained a 80 cm^3^ soil mixture (soil: vermiculite = 1:1); each pot contained 20 seedlings that continued to grow for 10 days under normal conditions. Drought was induced by not providing water to the 3-week-old plants after saturated water treatment by soil drenching. The control plants were well watered during this period. Physiological indices and chlorophyll fluorescence parameters were measured after 21 days of drought treatment. After 21 days drought stress, plants were watered and allowed to grow for 7 days.

### Measurements of physiology indices

#### MDA assay

MDA level was measured as described by Xing et al. with some modifications [25]. Fresh rosette leaves (0.1 g) from 5 to 6 different plants of the same *Arabidopsis* line were added to 2 mL of 10% (w/v) TCA, ground into a slurry, and then 8.0 mL of 10% TCA was added. The homogenate was centrifuged at 4000 rpm for 10 min. Two milliliters of the supernatant was then added to 2 mL of 0.6% (w/v) TBA, and 2 mL of distilled water was added to 2 mL of 0.6% TBA as blank control. The mixtures were heated at 100°C for 15 min and then quickly cooled in ice. After centrifugation at 4000 rpm for 10 min, the absorbance of the supernatant at 600, 532, and 450 nm was measured. MDA level was calculated using the absorption coefficient: MDA (mmd.g^-1^FW) = [6.452 × (A_532_-A_600_)-0.559 × A_450_] × V_t_/V_s_ × W.

Vt: The total extraction liquid volume 10.0 mL

Vs: The extraction liquid volume used for measurement 2.0 mL

W: Fresh weight of sample (g) 0.1g

This experiment was repeated 3 times for each line.

#### Relative membrane permeability

Leaf electrolytes in plants were measured using the protocol of Blum and Ebercon [26]. Leaves (0.1g) of similar sizes from 5 to 6 different plants of the same *Arabidopsis* line were briefly washed with distilled water and then immersed in a tube containing 15 mL of distilled water for 2 h of oscillation at 25°C. The electrical conductivity (C1) of the solution was then measured using a conductometer (Model DDS-11A; Shanghai Leici Instrument Inc., Shanghai, China). Samples were then heated in boiling water for 15 min and oscillated for 2 h. The conductivity of killed tissues (C2) was also measured. Relative membrane permeability RMP(%) = (C1/C2) × 100. This experiment was repeated 3 times for each line.

#### Measurement of free proline

Free proline content was measured spectrophotometrically according to the method of Bates et al [27]. Leaf tissues (0.1g) from 5 to 6 different plants of the same *Arabidopsis* line were homogenized in 5 mL of sulfosalicylic acid (3%), boiled in a water bath at 100°C for 15 min, and then cooled and centrifuged at 4000 rpm for 10 min. Two milliliters of the supernatant was then added to a test tube, and 2 mL of glacial acetic acid and 2 mL of ninhydrin reagent (25%) were added. The reaction mixture was boiled in a water bath at 100°C for 30 min. After cooling the reaction mixture, 4 mL of toluene was added, and the mixture was vortexed for 30 s; the upper phase containing proline was measured using a spectrophotometer (UV-2550, Shimadzu, Japan) at 520 nm by using toluene as the blank. The proline content (μg/g FW) was quantified using the ninhydrin acid reagent method with proline as the standard [3,27].

#### Determination of chlorophyll fluorescence parameters

Chlorophyll fluorescence was determined using Maxi-Imaging-PAM (Walz, Germany). Five or six plants of the same line were selected. After 20 min of dark treatment, the plants were exposed to a photon density of 81 μmol m^–2^ s^–1^ and excited every 20 s. Seventeen time points were used to obtain a stable status of the following parameters: effective PSII quantum yield [Y(II)], quantum yield of nonregulated energy dissipation in PSII [Y(NO)], electron transport rate (ETR), and quantum yield of regulated energy dissipation in PSII [Y(NPQ)].

#### Data analysis

Analysis of experimental data and generation of tables and graphs were done performed using Excel 2007. All data were the average values of three independent experiments. The analyses of differences were performed using SPSS 13.0. The significance level was *p* < 0.05, and the highly significant level was *p* < 0.001.

## Results

### Empty pcDNA_3_ vector, pcDNA_3_-CAS, and pcDNA_3_-OsCAS transfected into HEK293 cells

**Fig 1:**
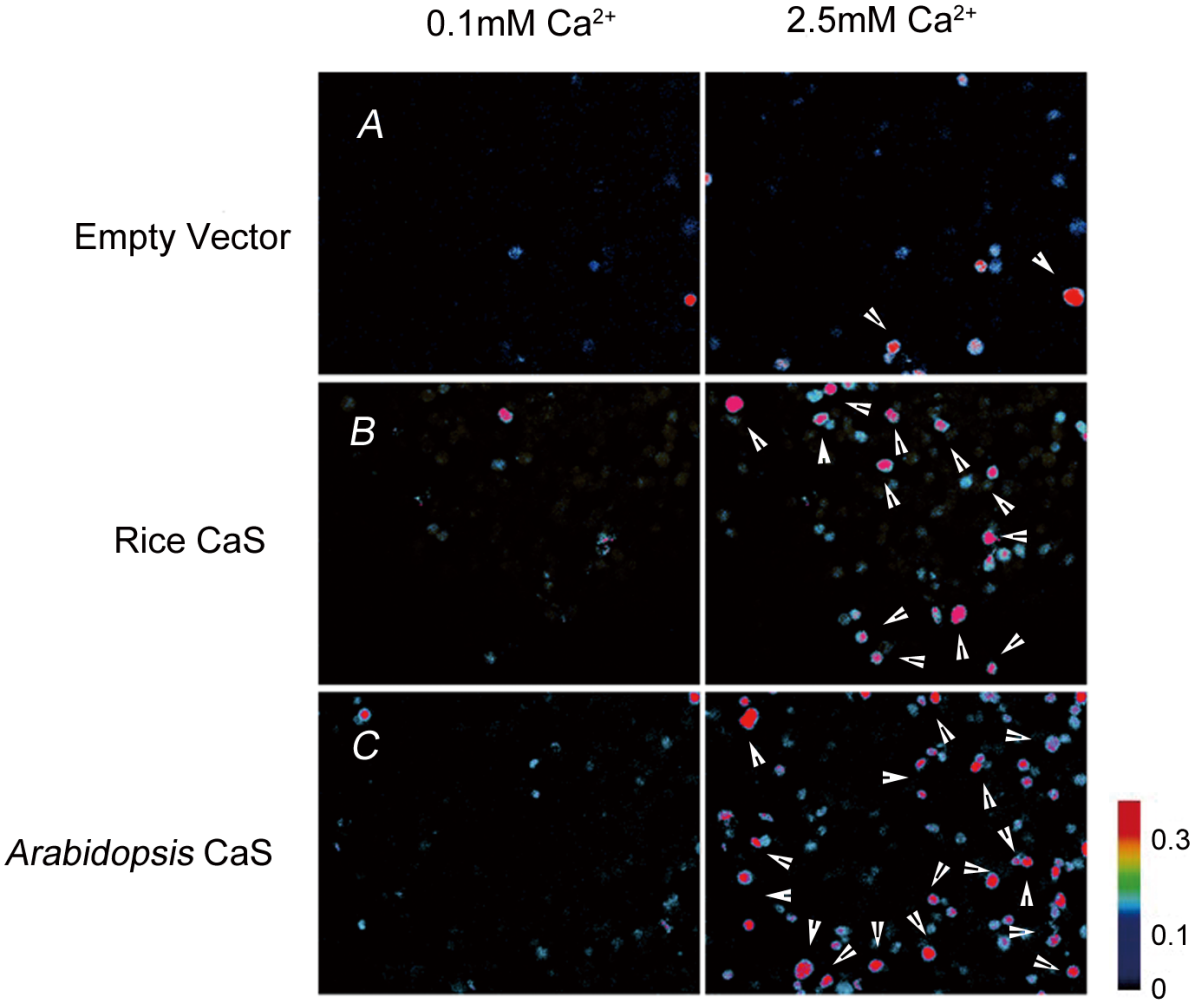
Expression cloning of rice CaS and *Arabidopsis* CaS using calcium imaging. HEK293 cells were transiently transfected with empty vector, pcDNA_3_-CAS, or pcDNA_3_-OsCAS. The cytosolic Ca^2+^ status was analyzed using Fura-2-based Ca^2+^ imaging after treatment with 0.l mM and 2.5 mM extracellular Ca^2+^. Elevated relative calcium concentrations are indicated by an increased ratio of Fura-2 emission at 340 versus 380 nm wavelength excitation (see color bar). Arrows show cells with CICI (Ca^2+^_o_-induced [Ca^2+^]_i_ increases).

HEK293 cells transiently transfected with vector alone (pCDNA_3_, Fig 1A) exhibited no response to extracellular Ca^2+^ increases from 0.1 to 2.5 mM. However, the rice CAS (Fig 1B) and *Arabidopsis* CAS (Fig 1C) were identified by marked increases in cytoplasmic calcium (arrowheads), which results in more cells sensitive to extracellular Ca^2+^.

### Screening and identification of transgenic plants

Transgenic plants were screened using hygromycin-selective medium. The positive plants grew true leaves and roots, while the negative plants developed into a rhizoid. Using the same method, homozygous plants of the T_3_ generation were screened: all seedlings could grow in hygromycin-selective medium without segregation, such as the lines L1, L2, L3, and L4.

**Fig 2:**
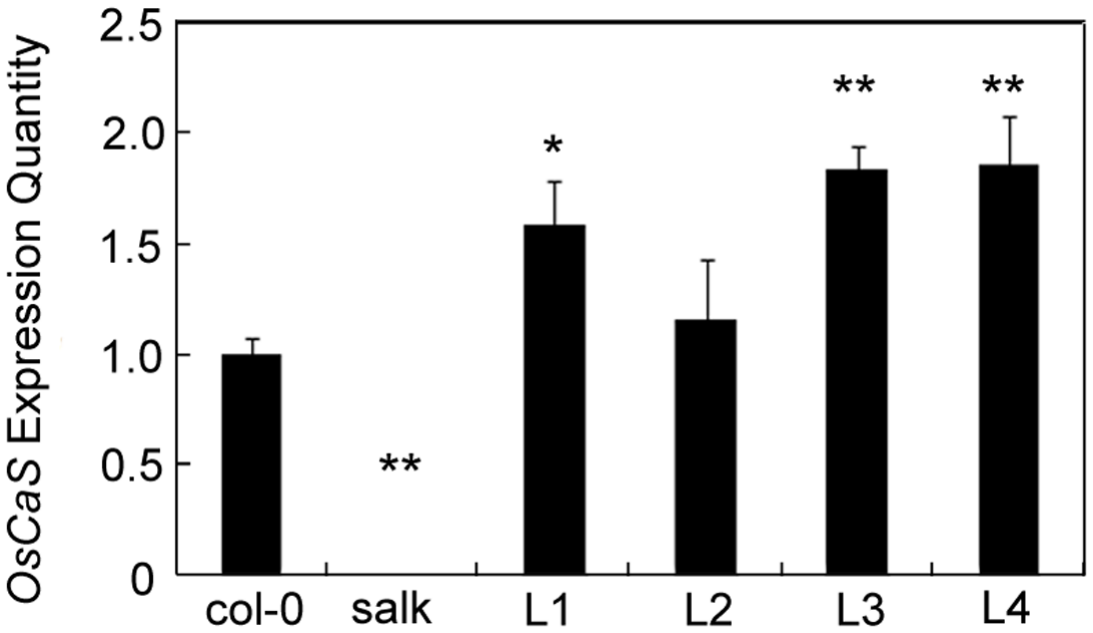
Detection of gene expression in transgenic plants by real-time PCR. Col-0 is the wild type; L1, L2, L3, and L4 represent *OsCAS* transgenic plants under the control of the CaMV 35S promoter; and Salk is the T-DNA insertion mutant. Mean and SE values were determined using at least three independent experiments. Significant differences from Col-0 control plants after drought treatment were determined by the t-test. * *p* < 0.05 and ** *p* < 0.01.

**Fig 3:**
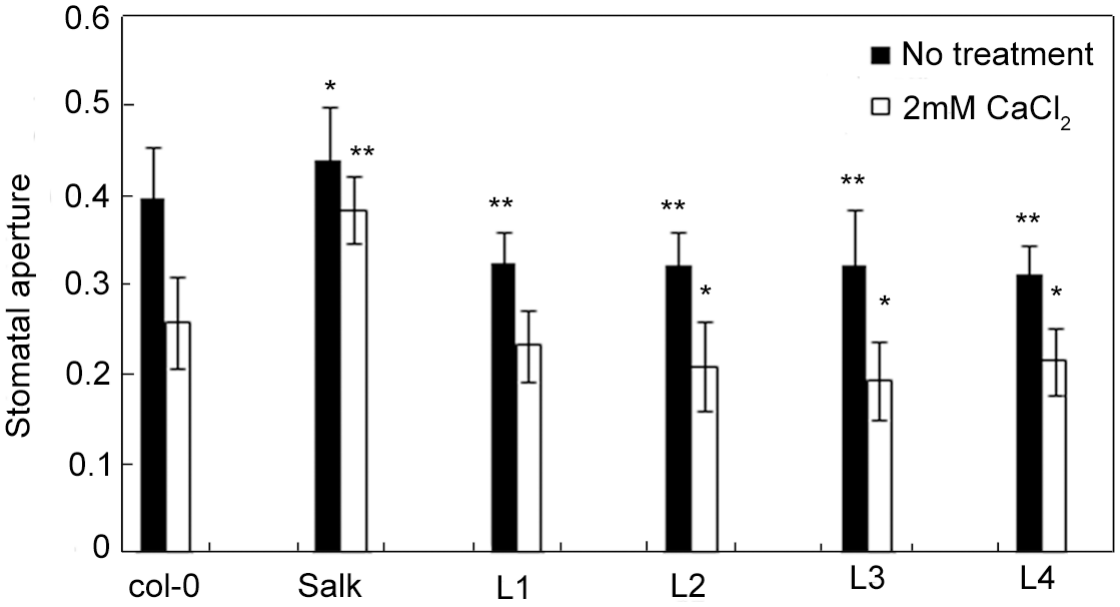
[Ca^2+^]_ext_-induced stomatal closure in mutant plants and transgenic lines. Stomatal aperture was calculated as the ratio of stomatal width to stomatal length. Each value is the mean of three independent measurements, and the error bars indicate SD. Significant differences from Col-0 control plants (No treatment, 2 mM CaCl_2_ treatment) after drought treatment were determined by the t-test. * *p* < 0.05 and ** *p* < 0.01.

PCR was used to amplify *Hyg* and *OsCAS* genes to identify transgenic plants. Positive plants showed both the 916-bp band of the *Hyg* gene and the 1164-bp band of the *OsCAS* gene. However, Col-0 and Salk did not show these bands. Finally, four lines (L1, L2, L3, and L4) were identified as positive *OsCAS* transgenic plants (S2 Fig). To further verify the *OsCAS* expression in transgenic plants, qRT analysis was performed. qRT analysis demonstrated that the *OsCAS* gene was overexpressed in the transgenic plants (Fig 2A). To examine the role of *OsCAS* in transgenic plants, we analyzed stomatal movement. Interestingly, we found that *OsCAS* overexpression in the transgenic plants promoted stomatal closure in the absence of external Ca^2+^ (Fig 3). These results indicated that *OsCAS* was successfully transformed and expressed in the mutants. For the sake of efficiency, we selected L3 as the representative of transgenic plants in the next experiment.

### *OsCAS* increases the tolerance of *Arabidopsis* to drought stress

**Fig 4:**
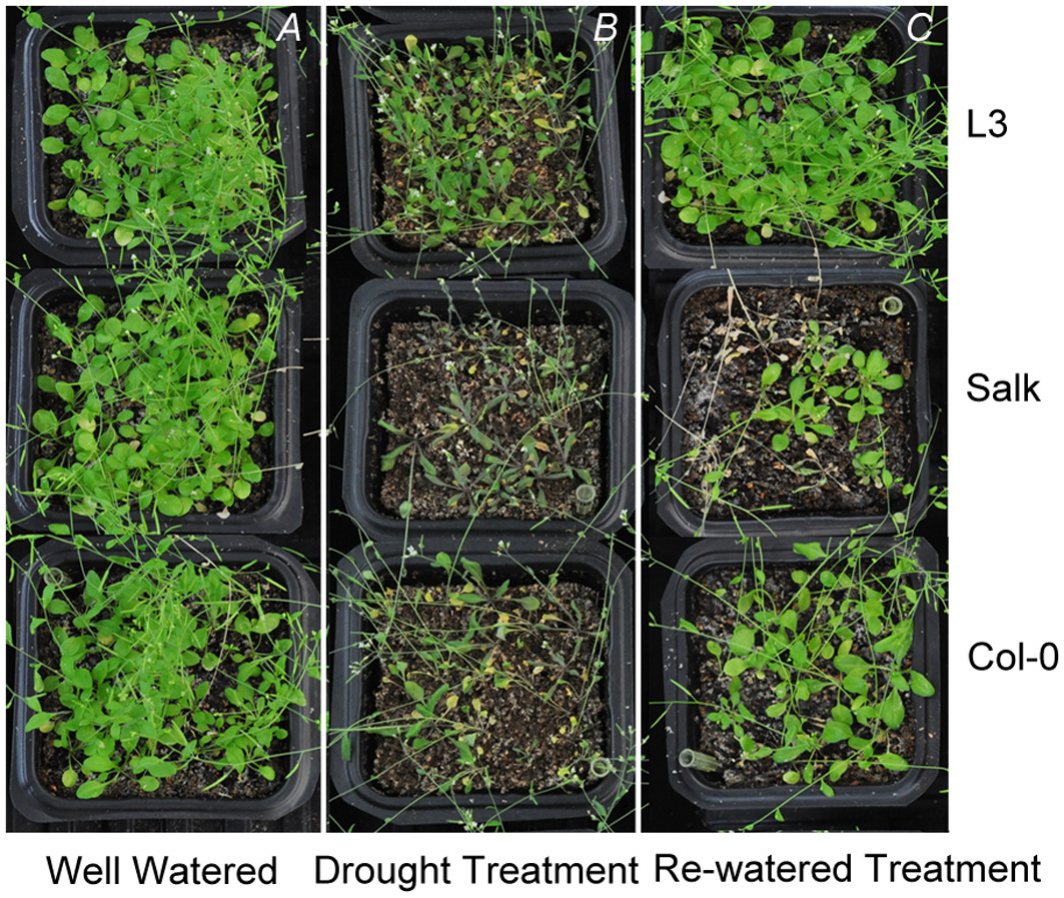
Drought stress tolerance of *OsCAS* transgenic plants. A. Plant growth under well-watered conditions. B. Plant growth under drought stress for 21 days. C. Plant re-watering treatment. In images A, B, and C, the first line of plants are *OsCAS* transgenic type; the second line of plants are Salk; and the third line of plants are wild-type Col-0.

Under well-watered conditions, there were no obvious differences in terms of appearance among Col-0, Salk, and transgenic line L3 (Fig 4A). After drought treatment for 21 days, the transgenic line L3 grew well and only some leaves turned yellow, whereas the leaves of the Col-0 and Salk plants showed severe wilting and curling (Fig 4B). When re-watered after drought treatment, some of the leaves turned green and the transgenic line L3 grew better than the Col-0 and Salk plants (Fig 4C). The survival rates of the transgenic line L3 after drought treatment were higher than those of the Col-0 and Salk plants (S3 Fig). These results indicated that expression of *OsCAS* increased the tolerance of *Arabidopsis* to drought stress.

**Fig 5:**
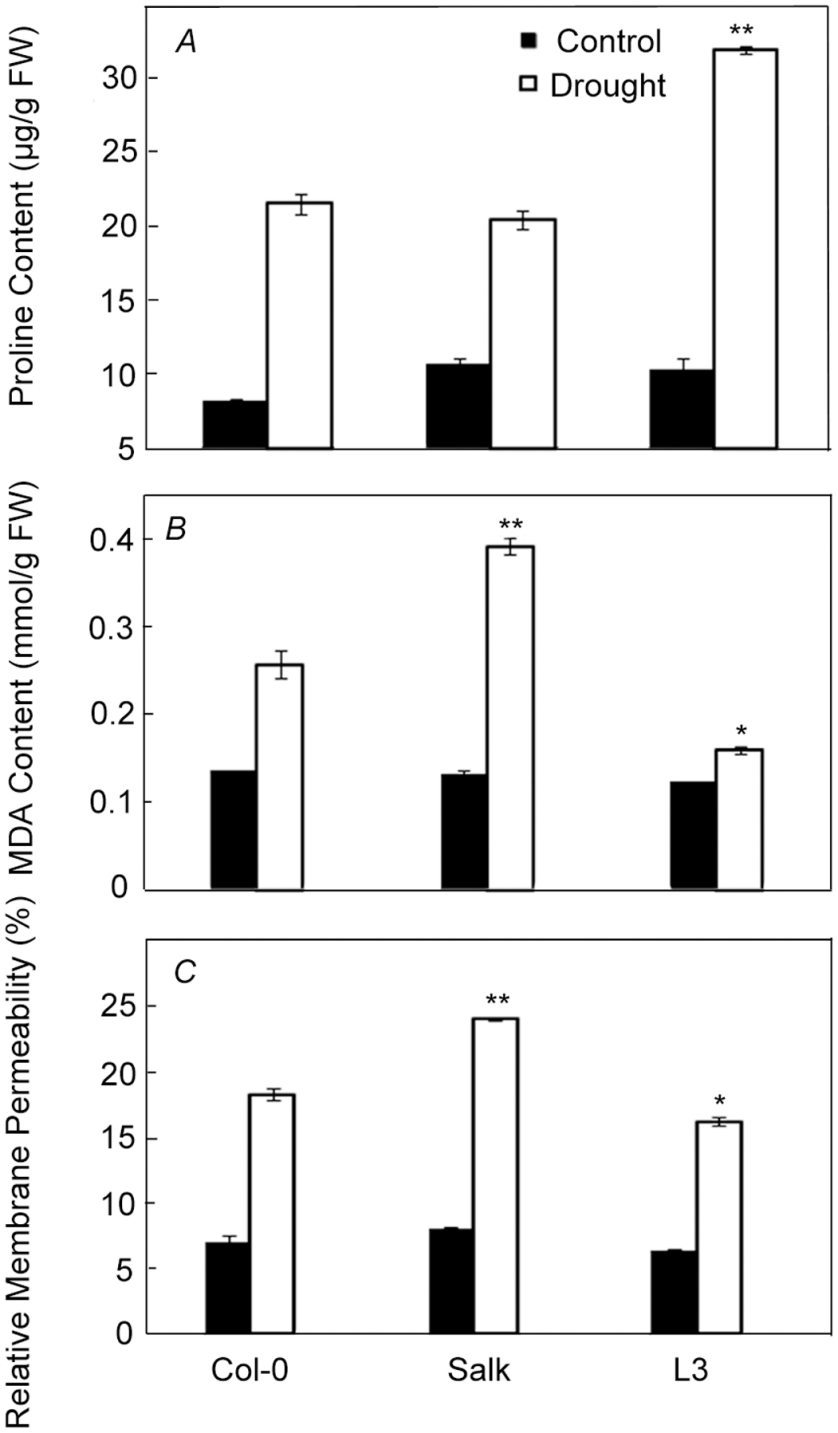
Free proline content, MDA content, and RMP in Col-0, Salk, and L3 after drought treatment. Each bar represents the mean from three replicates ± SD. Significant differences from Col-0 after drought stress were determined by the t-test. *p < 0.05 and**p < 0.01.

Physiological and biochemical parameters, including proline content, relative membrane permeability (RMP), and MDA content, were measured under normal growth and drought stress conditions. Proline, as an osmoprotectant, plays a critical role in protecting plants under drought stress conditions. Under normal growth conditions, the content of free proline was similar among Col-0, Salk, and transgenic line L3. However, after water stress for 21 days, the L3 transgenic line accumulated more free proline than Col-0 plants, while the Salk plants accumulated less (Fig 5A). To test membrane stability, RMP and MDA contents were determined. Under normal growth conditions, the RMP and MDA contents of transgenic line L3 were similar to those of Salk and Col-0 plants. Under drought stress treatment for 21 days, the RMP and MDA in Col-0 and Salk plants were greater than those in transgenic line L3; in particular, those of the Salk plants were significantly higher (Fig 5B and 5C). Thus, the degree of cell membrane damage in *OsCAS* transgenic plants was less than that in Col-0 and Salk plants under drought stress conditions.

Seven days after re-watering, the recovered leaves were used to test MDA, RMP, and proline content. The result showed that all the parameters were similar to those before drought treatment (S4 Fig).

**Fig 6:**
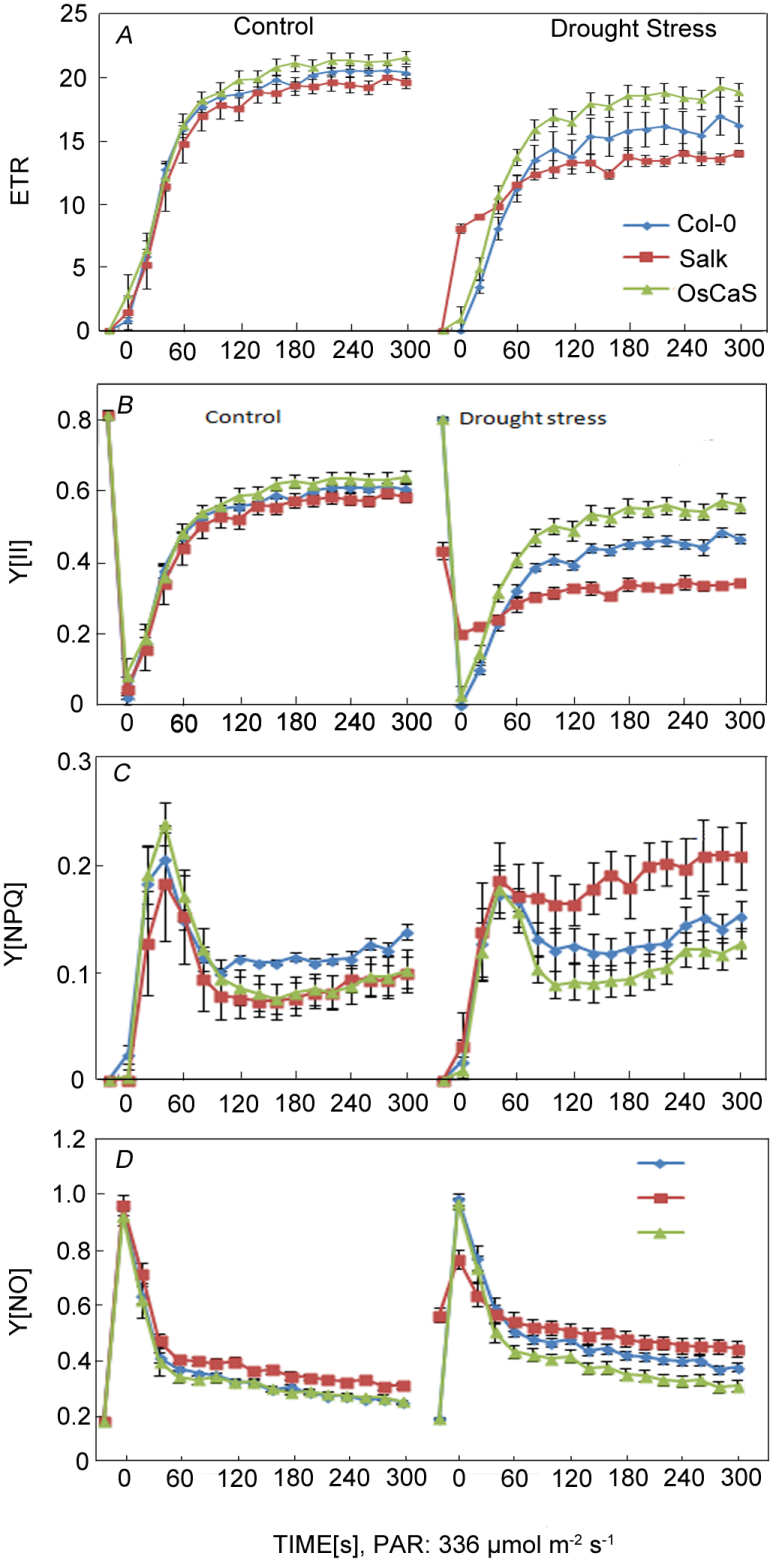
Chlorophyll fluorescence of Col-0, Salk, and L3 under normal and drought stress conditions. Chlorophyll fluorescence of the control was measured under normal conditions. Values represent the mean ± SD (n = 3, independent experiments). Values of ETR (A), Y(II) (B), Y(NPQ) (C), and Y(NO) (D) in the three lines.

Chlorophyll fluorescence parameters, including electron transport rate (ETR) and actual photosynthetic efficiency [Y (II)], were measured under well-watered and drought stress conditions. The ETR values of Col-0 and Salk decreased significantly after 3 weeks of drought, particularly in Salk (*p* < 0.01). However, the ETR values in *OsCAS* transgenic L3 plants remained relatively stable (Fig 6A). Changes in Y(II) values in the three lines were consistent with that of ETR under normal and drought stress conditions (Fig 6B). These changes indicated that the actual photosynthetic efficiency in the transgenic plants was higher than that in Salk and Col-0 under drought stress.

Y(NPQ) represents quantum yield of regulatory energy dissipation. Compared with normal conditions, the Y (NPQ) values of Salk increased significantly (*p* < 0.01), and those of L3 and Col-0 plants were relatively stable under drought stress (Fig 6C). This indicated that the expression of Os*CAS* could influence the regulation of energy dissipation and susceptibility to photoinhibition.

Y (NO) represents the quantum yield of nonregulated energy dissipation. Under the control conditions, the Y (NO) values were not significantly different in Col-0, Salk, and transgenic plant L3. Compared with control conditions, the Y (NO) values of all lines increased under drought stress. In addition, the Y (NO) values of Salk were always higher than those of the other lines under well-watered and drought stress conditions (Fig 6D). This indicated that all three lines had a certain degree of photo damage, and that in Salk was more severe than in line L3 under drought stress.

## Discussion

Previous studies have shown that CaS is localized mainly in chloroplasts and is a plant-specific Ca^2+^-binding protein. It is an integral thylakoid membrane protein and is essential for stomatal closure. T-DNA insertion in the *CaS* knockout mutants failed to control stomatal closure induced by external Ca^2+^ ions. In contrast, overexpression of CaS promoted stomatal closure in the absence of external Ca^2+^ ions [18]. The carboxy terminus of CaS has sequence similarity to rhodanese domain proteins, which are associated with specific stress conditions [13,20]. Our previous studies have shown that overexpression of *OsCAS* in rice could enhance the drought resistance of plants [28,29]. It has homologs in *Oryza sativa* and *Arabidopsis thaliana*, as well as some lower plants. *Arabidopsis thaliana* is often used as a plant model to study the physiological and biochemical characteristics of plants. In addition, it is an ideal model system used in the fields of genetics and molecular biology [20,30]. In order to further study the functions of *OsCAS*, we transformed *OsCAS* into an A*rabidopsis CaS* knockout mutant (Salk) and obtained four lines that overexpressed *OsCAS*. The results suggested that OsCAS overexpression could promote stomatal closure, which is consistent with previous results (Fig 3). Moreover, *OsCAS* transgenic plants showed significant drought tolerance. This indicates that the *OsCAS* gene is associated with drought stress tolerance.

Drought stress causes membrane lipid peroxidation, which results in the accumulation of MDA and changes in RMP levels in plants. Therefore, MDA and RMP levels have been used as an efficient indicator to evaluate the degree of the drought resistance [3,4,5]. Accumulation of osmoprotectants can reduce cellular osmotic potential so that plants can tolerate stress. Proline, an osmoprotectant that increases proportionately faster than other osmoprotectants in plants under drought stress, has been suggested as a parameter for selecting drought-resistant varieties [27]. Our results showed that RMP and MDA levels in the transgenic plants were lower, whereas proline content was higher, than in wild-type plants under drought stress (Figs 5A, 5B and 5C). These results suggest that the degree of membrane damage in transgenic plants was less than that in wild-type plants under drought stress.

The inhibition of photosynthesis and decrease of photosynthetic capacity are the primary physiological consequences of drought stress [31,32]. Chlorophyll fluorescence as a noninvasive method is widely used for the study of photosynthesis under stress [8,33,34]. In photosynthesis research, Y(II), Y(NPQ), and Y(NO) represent the quantum yield, and the regulated and nonregulated energy dissipation at PSII centers, respectively. The sum of all quantum yields is always equal to 1: Y(II)+Y(NPQ)+ Y(NO) = 1 [13]. In principle, a high Y(II) value means that more quanta absorbed by PSII are converted into chemically fixed energy; a high Y(NPQ) value indicates that the photon flux density is excessive and the plant has retained the physiological means to protect itself by regulation; and a high Y(NO) value indicates that both photochemical energy conversion and protective regulatory mechanisms are inefficient and indicates that the plant is already damaged or will be photodamaged [13,35]. The value of the ETR is that it can be used to assess the physiological state of plants [17,36]. In this study, our results showed that, compared with wild-type plants, Y (II) and ETR values were higher in *OsCAS* transgenic plants but lower in Salk plants under drought stress. In contrast, the Y (NPQ) and Y (NO) values were lower in transgenic plants but higher in Salk plants compared with those in the Col-0 controls under drought stress. These results indicated that *OsCAS* could increase the efficiency of photochemical energy conversion and decrease the photo-damage when plants are under drought stress.

In conclusion, *OsCAS* can decrease membrane damage and inhibition of photosynthesis under drought stress. Therefore, there is a close relationship between *OsCAS* and drought tolerance, that is, *OsCAS* can enhance the resistance of transgenic *Arabidopsis* to drought stress.

## Supporting Information

S1 Fig Sketch map of the 35S::*OsCAS*–GFP vector. (TIF)

S2 Fig PCR detection of transgenic plants. M: marker; W: Col-0; S: Salk mutant; H: H_2_O; P: positive control; L1, L2, L3, and L4 are homozygous transgenic plants. (a) PCR detection of the *OsCAS* gene in transgenic plants. (b) PCR detection of the *Hyg* gene in transgenic plants.
(TIF)

S3 Fig Statistics of survival rate after re-watering. Significant differences from control plants Col-0 were determined by the t-test after drought treatment. * *p* < 0.05 and ** *p* < 0.01.
(TIF)

S4 Fig Free proline content, MDA content, and RMP in Col-0, Salk, and L3 after re-watering. Each bar represents the mean from three replicates ± SD. Significant differences from Col-0 were determined by the t-test after drought stress. *p < 0.05 and**p < 0.01.
(TIF)

S1 Table Primers for PCR reaction. (DOCX)
