# Supplementary figures and images for: Expression of OsCAS (Calcium-Sensing Receptor) in an Arabidopsis Mutant Increases Drought Tolerance
Source: PLoS One. 2015 Jun 22;10(6):e0131272. doi: 10.1371/journal.pone.0131272 (PMC4476762; doi:10.1371/journal.pone.0131272)

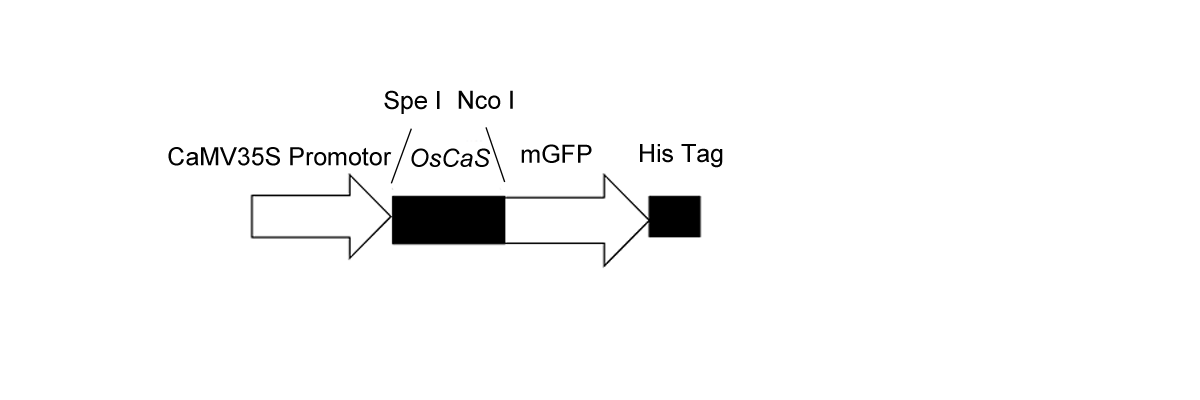

Supplement: S1 Fig — (TIF) [file pone.0131272.s001.tif]

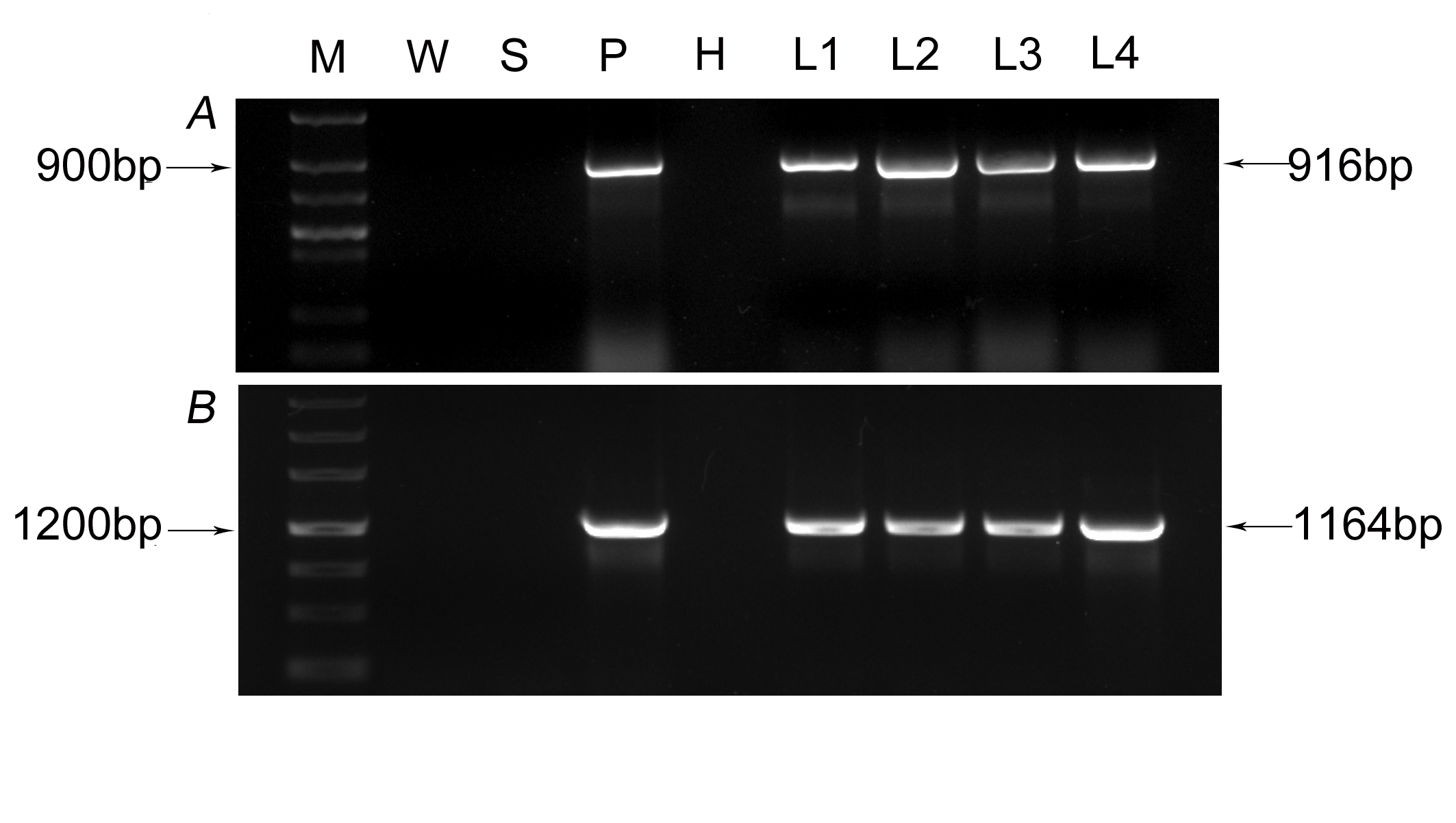

Supplement: S2 Fig — M: marker; W: Col-0; S: Salk mutant; H: H2O; P: positive control; L1, L2, L3, and L4 are homozygous transgenic plants. (a) PCR detection of the OsCAS gene in transgenic plants. (b) PCR detection of the Hyg gene in transgenic plants. (TIF) [file pone.0131272.s002.tif]

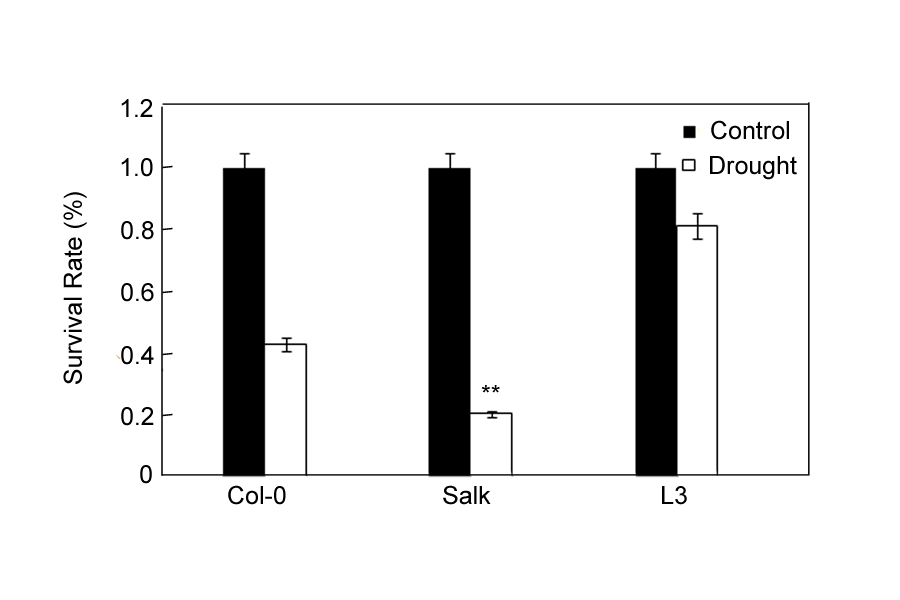

Supplement: S3 Fig — Significant differences from control plants Col-0 were determined by the t-test after drought treatment. * p < 0.05 and ** p < 0.01. (TIF) [file pone.0131272.s003.tif]

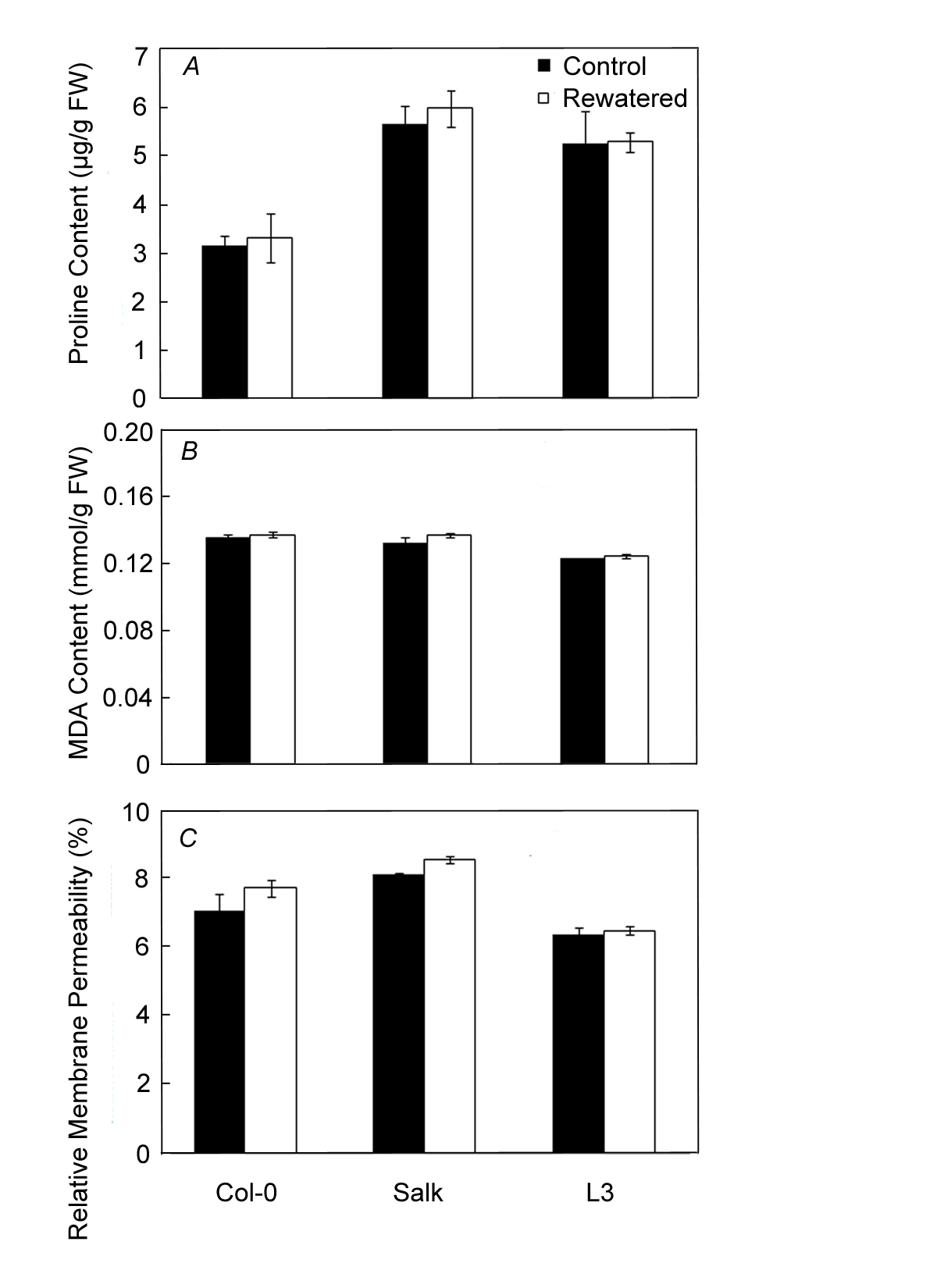

Supplement: S4 Fig — Each bar represents the mean from three replicates ± SD. Significant differences from Col-0 were determined by the t-test after drought stress. *p < 0.05 and**p < 0.01. (TIF) [file pone.0131272.s004.tif]
