# Supplementary material for: Expression of OsCAS (Calcium-Sensing Receptor) in an Arabidopsis Mutant Increases Drought Tolerance
Source: PLoS One. 2015 Jun 22;10(6):e0131272. doi: 10.1371/journal.pone.0131272 (PMC4476762; doi:10.1371/journal.pone.0131272)
Supplement: S1 Table — (DOCX) [file pone.0131272.s005.docx]

**S1 table. Primers for PCR reaction.**

| Gene | Primer sequence F^a^/R^b^ (5′-3′) |
| --- | --- |
| (1) *OsCAS* for PCR | CATGCCATGGCCATGGCGCCCCTTTCG/GGACTAGTGCCGTCCACGCTGCC |
| (2) Hyg for PCR | AAGTTCGACAGCGTCTCCGAC/TCTACACAGCCATCGGTCCAG |
| (3) *AtCaS* for qRT-PCR | GGTGCTGCGTTTCTTGCTTACC/AGCCGGCGTAAGATCACCTTTG |
| (4) *OsCAS* for qRT-PCR | TGGTCCCTGCTTTCTTTCACTC/CTCCGCCTCGGCTTGTTT |
| (5) Actin of *Arabidopsis* | CACTGTGCCAATCTACGAGGGT/CACAAACGAGGGCTGGAACAAG |

**^a^**forward,  ^b^reverse
